# Human Osteoarthritic Cartilage Shows Reduced *In Vivo* Expression of IL-4, a Chondroprotective Cytokine that Differentially Modulates IL-1β-Stimulated Production of Chemokines and Matrix-Degrading Enzymes *In Vitro*

**DOI:** 10.1371/journal.pone.0096925

**Published:** 2014-05-12

**Authors:** Elisa Assirelli, Lia Pulsatelli, Paolo Dolzani, Daniela Platano, Eleonora Olivotto, Giuseppe Filardo, Giovanni Trisolino, Andrea Facchini, Rosa Maria Borzì, Riccardo Meliconi

**Affiliations:** 1 Laboratory of Immunorheumatology and Tissue Regeneration/RAMSES, Rizzoli Orthopaedic Institute, Bologna, Italy; 2 Laboratory of Biomechanics and Technology Innovation, 2^nd^ Orthopaedic and Traumatologic Clinic, Rizzoli Orthopaedic Institute, Bologna, Italy; 3 Reconstructive Hip and Knee Joint Surgery, Rizzoli Orthopaedic Institute, Bologna, Italy; 4 University of Bologna, Bologna, Italy; 5 Medicine and Rheumatology Unit, Rizzoli Orthopaedic Institute, Bologna, Italy; National Center for Scientific Research Demokritos, Greece

## Abstract

**Background:**

In osteoarthritis (OA), an inflammatory environment is responsible for the imbalance between the anabolic and catabolic activity of chondrocytes and, thus, for articular cartilage derangement. This study was aimed at providing further insight into the impairment of the anabolic cytokine IL-4 and its receptors in human OA cartilage, as well as the potential ability of IL-4 to antagonize the catabolic phenotype induced by IL-1β.

**Methodology/Principal Findings:**

The *in vivo* expression of IL-4 and IL-4 receptor subunits (IL-4R, IL-2Rγ, IL-13Rα1) was investigated on full thickness OA or normal knee cartilage. IL-4 expression was found to be significantly lower in OA, both in terms of the percentage of positive cells and the amount of signal per cell. IL-4 receptor type I and II were mostly expressed in mid-deep cartilage layers. No significant difference for each IL-4 receptor subunit was noted. IL-4 anti-inflammatory and anti-catabolic activity was assessed *in vitro* in the presence of IL-1β and/or IL-4 for 24 hours using differentiated high density primary OA chondrocyte also exhibiting the three IL-4 R subunits found *in vivo*. Chemokines, extracellular matrix degrading enzymes and their inhibitors were evaluated at mRNA (real time PCR) and protein (ELISA or western blot) levels. IL-4 did not affect IL-1β-induced mRNA expression of GRO-α/CXCL1, IL-8/CXCL8, ADAMTS-5, TIMP-1 or TIMP-3. Conversely, IL-4 significantly inhibited RANTES/CCL5, MIP-1α/CCL3, MIP-1β/CCL4, MMP-13 and ADAMTS-4. These results were confirmed at protein level for RANTES/CCL5 and MMP-13.

**Conclusions/Significance:**

Our results indicate for the first time that OA cartilage has a significantly lower expression of IL-4. Furthermore, we found differences in the spectrum of biological effects of IL-4. The findings that IL-4 has the ability to hamper the IL-1β-induced release of both MMP-13 and CCL5/RANTES, both markers of OA chondrocytes, strongly indicates IL-4 as a pivotal anabolic cytokine in cartilage whose impairment impacts on OA pathogenesis.

## Introduction

A complex interplay of biomechanical, metabolic and biochemical factors have been thought to contribute to progressive cartilage damage in OA by inducing and maintaining an imbalance between the degradation and synthesis rate of the extracellular matrix component [1]. IL-1β is known as a major inducer of articular cartilage extracellular matrix (ECM) breakdown, promoting the production and activation of different factors that act as mediators and/or effectors of progressive cartilage loss [1].

Chemokines, such as MCP-1/CCL2, RANTES/CCL5, GROα/CXCL1, IL-8/CXCL8, MIP-1α/CCL3 and MIP-1β/CCL4, actively take part in these catabolic pathways [2], by locally amplifying a catabolic microenvironment and the effects of the microenvironment on extracellular matrix-degrading enzymes. These enzymes include metalloproteinases (MMPs) and aggrecanases (a Disintegrin and Metalloproteinase with Thrombospondin Motifs-ADAMTS), which act as down-stream key players in the inflammatory signal cascade together with their soluble inhibitors (Tissue Inhibitors of Metalloproteinases-TIMPs) [3].

However, the expression of anabolic growth factors and anti-catabolic cytokines occurs in OA cartilage [1], thus showing the ability of chondrocytes to set-up compensatory, yet ineffective, mechanisms to counteract cartilage breakdown. Among these soluble factors is IL-4, whose function of maintaining cartilage homeostasis has been previously highlighted. IL-4 has a potent anti-inflammatory action able to inhibit the synthesis of IL-1β and TNFα [4], [5] and down regulate many effects of these cytokines. Indeed, IL-4 is able to down regulate the IL-1β-induced production of several mediators of cartilage breakdown (e.g.: nitric oxide, prostaglandins, MMPs) [6], [7] and to reverse IL-1β inhibition of proteoglycan synthesis [8]. In addition, the local over-expression of IL-4 protected cartilage from matrix metalloproteinase-induced destruction during experimental immune complex-mediated arthritis and the intra-articular injection of IL-4 ameliorate the cartilage destruction in instability-induced OA model in rat knee joints [9]. Very recently, the synergic chondroprotective activity of IL-4 in conjunction with IL-10 has been reported [10], [11]. Most of these results, which support the potential chondroprotective/anti-catabolic effect of IL-4, have mainly been obtained using animal models in *‘in vivo’* or *‘in vitro’* studies, but little information is currently available concerning the IL-4 modulation of IL-1β-induced response in human articular chondrocytes.

The majority of studies addressing the role of IL-4 in human cartilage have focused on its involvement in the mechanotransduction pathway [12], [13], [14]. Mechanotransduction of chondrocytes triggers a signalling cascade that induces IL-4, a major active autocrine/paracrine signalling molecule [13], [14], [15] able to induce an anabolic response of normal chondrocytes, which appears to be impaired in OA cartilage [13].

Proteomic and genomic studies support evidence that anabolic activity continues in OA chondrocytes, but their ability to restore the integrity of cartilage matrix appears to be impaired [16]. Nevertheless, several studies have previously suggested the beneficial and anti-inflammatory effects of biomechanical signals in the physiological range in the management of arthritic joints [17].

IL-4 induces its cellular responses by binding to a multimeric receptor complex, the IL-4 receptor (IL4-R). IL4-R consists of a primary IL-4Rα subunit, which can dimerize either with a common gamma chain (γc or IL-2R) to form a Type I receptor or with an IL-13Rα' subunit (also known as IL-13Rα1) to form a Type II receptor [7], [18], [19]. Binding IL-4 to an IL4-Rα subunit leads to multimeric receptor complex formation and the subsequent activation of the Janus Kinase/signal transducers and activator of transcription (JAK/STAT) pathway [20].

IL-4 relevance in OA is also supported by genetic evidence, as highlighted by the association of the IL-4 receptor (IL-4R) gene polymorphisms observed in different subsets of OA [21], [22]. In addition, our previous study showed a differential systemic modulation of soluble IL-4 receptor levels in different OA subsets compared to normal subjects [23]. In this study, we investigated the differential protein expression of IL-4 and its receptors in OA and normal cartilage, as well as IL-4 modulating activity on IL-1β catabolic effects. In particular, we evaluated whether IL-4 was able to affect the IL-1β-induced production of chemokines (CXC chemokines: GROα/CXCL1, IL-8/CXCL8 and CC chemokines: RANTES/CCL5, MIP-1α/CCL3, MIP-1β/CCL4), ECM degrading enzymes (MMP-13, ADAMTS-4, ADAMTS-5) and their inhibitors (TIMP-1 and -3) in human OA chondrocytes.

Among several potential inflammatory/catabolic mediators, chemokines were selected because of their relevance in OA pathophysiology [2],[24] and because, due to their small size, they can greatly contribute to the crosstalk between joint compartments (cartilage, synovium and subchondral bone) [25]. The two ADAMTS and MMP-13 were selected because they are pivotal matrix-degrading enzymes in OA disease, being responsible for the initial aggrecan and collagen 2 cleavage, respectively, the main components of cartilage extracellular matrix [26].

Our aim was to determine the molecular reasons for the impairment of IL-4 anti-catabolic activity in osteoarthritis. We found that IL-4 is able to abrogate the IL-1β-dependent induction of RANTES and MMP-13, at both RNA and protein levels, and since we also found that IL-4 expression is reduced in OA cartilage, we hypothesize that the overall reduction of this anti-catabolic/anti-inflammatory activity plays a pivotal role in the functional impairment of osteoarthritic cartilage.

## Materials and Methods

All the data analyzed in the manuscript will be made freely available upon request for the purpose of academic, non-commercial research.

### Ethics statement

The study was approved by the ethical committee of the Rizzoli Orthopaedic Institute and written informed consent was obtained from all patients. Patient names were replaced by arbitrary codes for the sake of anonymity.

### IL-4 and IL-4R expression on cartilage samples

#### Patients

IL-4 and IL-4R subunit (IL-4Rα chain, IL-13Rα1 chain and common γ chain) expression was investigated in full thickness cartilage specimens of samples derived from either normal cartilage (5 non-OA cartilage samples derived from amputation due to oncological diseases, mean age 43, range 7-69) (NC) or from 6 OA patients (mean age 66.7 years, range 59-76) (OA) undergoing knee replacement. The grading of OA cartilage samples undergoing analysis was 1-2 according to published criteria [27]. More in detail, immunohistochemistry was used to assess the zonal distribution of each of the IL-4R subunits across cartilage, and the percentage of positive cells in either the superficial or the mid-deep layers. Immunofluorescence, coupled with confocal microscopy analysis, was instead required to assess IL-4 expression at the single cell level in order to provide quantitative information and compare NC and OA cartilage. Furthermore, immunofluorescence was also used to assess the colocalization (highly suggestive of dimerization) of IL-4Rα1 with either IL-2Rγ chain or IL-13Rα1.

The diagnosis of OA was based on clinical, laboratory and radiologic evaluations [28]. Full thickness cartilage specimens collected as described in [29] were snap-frozen and stored at −80°C until processing to obtain serial cryostat sections (5 µm thick) for immunohistochemistry or confocal microcopy performed as detailed below.

#### Immunohistochemistry

Immunohistochemistry was used to evaluate the percentage of IL4-R subunits (IL-4Rα chain, IL-13Rα1 chain and common γ chain) positive cells across cartilage. Defrosted sections were fixed in 4% paraformaldehyde at room temperature for 30 minutes, rehydrated and incubated overnight at 4°C with primary antibodies to the IL-4Rα chain (mouse monoclonal MAB230, IgG2a R&D Systems) at a concentration of 10 µg/ml, to the IL-13Rα1 chain (Goat AF152 R&D Systems) at a concentration of 1 µg/ml and to a common gamma chain (Goat AF284 R&D Systems) at a concentration of 1 µg/ml. Signals were developed with a biotin/streptavidin amplified, alkaline phosphatase-based detection system (SuperSensitive Link, Biogenex, San Ramon, CA) with fuchsin as a substrate. For Goat polyclonal antibodies, biotinylated bovine anti-Goat secondary antibody (Jackson Laboratories) was used at 1 µg/ml.

After nuclear counterstaining with hematoxylin, sections were mounted in glycerol gel and stored for subsequent analysis. Sections of each biopsy were processed as negative controls according to the above-described procedure but the primary antibody was omitted. Specificity control was assessed using mouse IgG2a isotype control (R&D Systems) or Normal Goat Ig at the concentration of the corresponding primary antibody.

Results of the immunohistochemical analysis were expressed as percentages of cells expressing each of the three IL-4R subunits over the total number of chondrocytes.

#### Immunofluorescence and confocal microscopy analysis

Immunofluorescence and confocal microscopy analysis was used to compare cellular IL-4 content in NC (n = 3) and OA (n = 6) cartilage samples. In a subset of these samples, the colocalization of the various IL-4R subunits (IL-4Rα chain, IL-13Rα1 chain and common γ chain) across cartilage layers was also investigated. Frozen 5- µm cartilage sections from explants were fixed with 4% PFA, unmasked with 0.02 U/ml chondroitinase for 20 min at 37°C, and blocked with 5% bovine serum albumin (BSA) in 0.1% Triton in TBS for 30 min at room temperature; then sections were incubated overnight at 4°C with primary antibodies in TBS with 3% BSA and 0.1% Triton. Antibodies against IL-4 (mouse monoclonal MAB304, IgG1 R&D Systems), IL-4 Rα mouse monoclonal MAB230, IgG2a R&D Systems), IL-13Rα1 (Goat AF152 R&D Systems) and IL-2Rγ (Goat AF284 R&D Systems) were used at concentrations of 40, 5, 12.5, and 12.5 µg/ml, respectively. Secondary antibodies: i) biotinylated bovine anti-goat IgG (3 µg/ml, Jackson ImmunoResearch Laboratories, Inc.) followed by streptavidin Alexa Fluor 555 (5 µg/ml, Molecular Probes, Eugene OR) for IL-13Rα1 and IL-2Rγ or ii) Donkey anti-mouse Dy Light 647 (15 µg/ml, Jackson ImmunoResearch Laboratories, Inc.) for IL-4 Rα were diluted in TBS with 3% BSA and 0.1% Triton and applied to tissue sections for 30 min at RT. After washing, the sections were incubated for 15 min with Sybr green (1∶10,000 fold dilution) (Molecular Probes, Eugene OR) for nuclear staining, and finally mounted with DABCO 90% glycerol. Isotypic controls were run in each experiment. Fluorescent signals were acquired by NIKON confocal microscope system A1 equipped with a Nikon Eclipse T*i* microscope and an Argon Ion laser for a 488 nm line, a DPSS laser for a 561 nm line and a diode laser for a 640 mn line. Emission signals were detected by a photomultiplier tube (DU4) preceded by emission filters BP 525/50 nm, BP 595/50 nm and BP 700/75 nm for Sybr green, Alexa Fluor 555 or Dy Light 647, respectively. Laser scanning, image acquisition and processing were performed with Nikon Imaging Software NIS Elements AR-4 (Nikon Inc., USA). Fields of 210 µm×210 µm (acquired with a Nikon plan apo 60×1.40 oil objective) were acquired and analyzed. Optical sections were spaced along the z-axis of approximately 0.5 µm for IL-4 quantification and 0.2 µm for receptor subunit colocalization, and were digitized with a scanning mode format of 512×512 pixels and 4096 gray levels.

To quantify IL-4 signal, for each patient, six to ten 60× fields (210 µm×210 µm) were acquired and analyzed. To avoid differences in staining intensity due to experimental conditions, all sections were processed in the same session of immunostaining and subsequently acquired in confocal microscopy using the same laser settings. The analytical power of confocal microscopy was fully exploited for IL-4 assessment, and therefore not only was the signal intensity taken into account, but also the extension of the positive area in the single cells. IL-4 signals were reported as Mean Intensity (i.e. mean of intensity values of pixels, NIS Elements AR2 Image Analysis) per cell positive area.

### IL-4 activity on IL-1β-induced chondrocyte production of chemokines, matrix-degrading enzymes and TIMPS

The functional role of IL-4 in modulating IL-1β activity was assessed using primary chondrocyte cultures established according to an *in vitro* high-density culture model [30] which was tested to assess: 1) the maintenance of a properly differentiated phenotype; 2) the expression of IL-4 receptor subunits at the protein and RNA level.

#### Patients

Isolated chondrocytes were obtained from cartilage derived from a total of 11 patients with knee OA (mean age 65.1 years, range 55–80) undergoing joint replacement surgery. The diagnosis of OA was based on clinical, laboratory and radiological evaluations [28]. All cartilage samples were obtained from tibial plateaus or femoral condyles.

The study was approved by the ethics committee of the Rizzoli Orthopaedic Institute and written informed consent was obtained from all patients.

#### Chondrocyte isolation, culture, and phenotype assessment

During tissue sampling, regions with signs of erosion were avoided and therefore the grading of the tissue samples utilized was 1–2 [27]. Cartilage was fragmented before digestion and chondrocytes were isolated by sequential enzyme digestion as previously detailed [31]. High-density chondrocyte cultures were obtained according to [30]. Briefly, cells were seeded at 62,500 cells per cm^2^ in 12-well plates. DMEM medium supplemented with 10% FBS was replaced every 2 days. Cultures reached confluence 5–7 days after plating and were then left in culture for an additional 7 days before cytokine stimulation.

The recovery of a differentiated phenotype in high-density cultured chondrocytes that had been previously expanded in culture was evaluated by probing total cellular lysates for SOX-9 (rabbit polyclonal AB5535 Millipore) expression, using GAPDH (mouse monoclonal MAB374 Millipore) Systems as a loading control. Western blot was performed as described in [29] in 4 different samples. In one of these samples, SOX-9 expression was compared in low-density-proliferating versus high-density-quiescent chondrocytes, loading a cell equivalent of 100,000 cells.

The IL-4 receptor subunit repertoire was assessed both at mRNA and protein levels. IL-4R subunits mRNA levels were evaluated by Real Time PCR in cDNA obtained from 5 unstimulated cultures, as described below (see Table 1 for primers sequence). Analysis of IL-4R subunits was also undertaken at the level of protein expression, both by flow cytometry to provide a quantitative assessment and by confocal microscopy, to provide information concerning the association of selected subunits. Flow cytometry was performed with early passage high density OA chondrocytes, recovered by trypsinization and then fixed with 2% PFA. The antibodies used were the same as those used for confocal analysis, IL-4Rα (Mouse R&D MAB230), IL-13Rα1 (Goat R&D AF152), IL-2Rγ (Goat R&D AF284) used at 10 µg/ml and detected by Donkey anti-Mouse Dy Light 647 or Donkey anti-Goat Dy Light 647 at 15 µg/ml. Control antibodies were either isotype control IgG2a or normal goat IgG.

**Table 1:** Primers for PCR analysis.

**GAPDH**	F(5′-CCTGGCCAAGGTCATCCATG-3′)
(NM_001289746)	R(5′CGGCCATCACGCCACAGTT-3′)
**IL-4Rα**	F(5′-TGGGCGTCAGCGTTTCCTGC -3′)
(NM_000418)	R(5′-CTGCGGGCTGGGTTGGGAAT-3′)
**IL-13Rα1**	F(5′-GGAATCCACCCGAGGGAGCCA-3′)
(NM_001560)	R(5′-ACGTCAATCACAGCAGACTCAGGAT-3′)
**IL-2R**	F(5′-CTGGAACGGACGATGCCCCG
(NM_000206)	R(5′-GGGCCCCTCCTTTTGGGGGA-3′)
**IL-8/CXCL8**	F(5′-ATGACTTCCAAGCTGGCCGTG-3′)
(NM_000584)	R(5′TTATGAATTCTCAGCCCTCTTCAAAAACTTCTC-3′)
**GROα/CXCL**	F(5′-ACTGAACTGCGCTGCCAGTG-3)’
(NM_001511)	R(5′-GGCATGTTGCAGGCTCCTCA-3′)
**RANTES/CCL5**	F (5′-AGGTACCATGAAGGTCTCC-3′)
(NM_001278736)	R(5′-GACTCTCCATCCTAGCTCA-3′)
**MIP-1α/CCL3**	F(5′-GAATCATGCAGGTCTCCAC-3) ′
(NM_002983)	R(5′-CGAAGCTTCTGGACCCCTC-3′)
**MIP-1β/CCL4**	F(5′-ACCATCAAGCTCTGCGTGACTG-3′)
(NM_002984)	R(5′-GCAGGTCAGTTCAGTTCCAGGTC-3′)
**MMP13**	F(5′-TCACGATGGCATTGCT-3′)
(NM_002427)	R(5′-GCCGGTGTAGGTGTAGA-3′)
**TIMP-1**	F(5′-CACCAAGACCTACACTG-3′)
(NM_003254)	R(5′-GTGACGGGACTGGAAG-3′)
**TIMP-3**	F(5′-CCTTGGCTCGGGCTCATC-3′)
(NM_000362)	R(5′-GGATCACGATGTCGGAGTTG-3′)

ADAMTS-4 and ADAMTS-5 were purchased from Qiagen (Hilden, Germany) (Cat n°PPH00889E, PPH14490A and PPH09588A).

#### Chondrocyte stimulation

Preliminary dose-response experiments were performed to optimize IL-1β and IL-4 concentrations.

The cytokine concentrations and incubation time were based on results obtained in previous dose-dependence and kinetic experiments to assess the optimal conditions for detecting chemokine production.

After 24 h starvation in medium without FBS, cultures were stimulated with 2 ng/ml of rhIL-1β (R&D Systems), 10 ng/ml of rhIL-4 (R&D Systems) or a mix of IL-1β and IL-4 at the same final concentration. After 24 h incubation, supernatants were collected and maintained at −80°C until their use in the ELISA test and cells were lysed for RNA extraction. This time point was selected on the basis of previous reports [32] stating its adequacy for the appreciation of IL-1β-dependent mRNA increase of chemokines and matrix-degrading enzymes, despite their different induction kinetics. Conversely, it proved to be adequate to appreciate protein modulation.

#### Real-time quantitative reverse transcriptase polymerase chain reaction (RT-PCR)

Total RNA was isolated using TRIZOL reagent (Invitrogen) following the protocol recommended by the manufacturer. RNA was reverse-transcribed using SuperScript First-Strand kit (Invitrogen).

RNA specific primers for PCR amplification (Table 1) were generated from GeneBank sequences using the LightCycler Probe Design Software (Roche). Real-time PCR was run on the LightCycler Instrument (Roche) using the QuantiTect SYBR Green PCR kit (QIAGEN, Hilden, Germany) and the increase in PCR product was monitored for each amplification cycle by measuring the increase in fluorescence due to the binding of SYBR Green I Dye to dsDNA. The crossing point values were determined for each sample and specificity of the amplicons was confirmed by melting curve analysis. Amplification efficiency of each amplicon was evaluated using 10-fold serial dilutions of positive control cDNAs and calculated from the slopes of log input amounts plotted versus crossing point values. They were all confirmed to be high (>92%) and comparable; mRNA levels for each target gene were calculated, normalized to glyceraldehyde-3 phosphate dehydrogenase (GAPDH) reference gene, according to the ΔΔCt method and expressed as “Number of molecules per 100,000 GAPDH”.

#### Measurement of chemokine and matrix-degrading enzyme levels

Chemokine concentrations in cell culture supernatants were evaluated using commercial DuoSet ELISA kits (R&D Systems) following the manufacturer's instructions.

MMP-13 protein levels in chondrocyte culture supernatants were measured by ELISA as previously described [33] with an assay able to detect both the pro-enzyme and the active form. ADAMTS-4 protein concentration was measured on cell lysates obtained by scraping the cultures together with the re-synthesized extracellular matrix since ADAMTS possess two unique thrombospondin (TS) type I motifs which are used by this class of enzymes to bind the extracellular matrix. Cell lysates and western blotting was performed as previously described [34] using a Rabbit antiserum (AB19166) to detect ADAMTS4, with mouse β-TUBULIN as a loading control.

### Statistical analysis

Data distribution did not fulfill the hypothesis of normality and variance equality and therefore non-parametric tests were used for comparisons.

Comparison of IL-4 intensity, percentage of positive cells for IL-4 or IL-4 receptor subunits in normal versus OA cartilage was performed with unpaired data analysis using the Mann-Whitney U test. Due to limited variability, IL-4 data were represented as mean ± S.E.M while IL-4R subunits data were reported as median and interquartile range according to the distribution of data.

mRNA or protein level of chemokines and matrix-degrading enzymes in unstimulated or IL-1β, IL-4 or IL-1β+IL-4-stimulated conditions were also reported as median and interquartile range and compared with non-parametric paired data analysis using Wilcoxon's test with Bonferroni's correction for multiple comparisons (value of p<0.0165 was considered significant after Bonferroni's correction). Statistical analysis was carried out using GraphPad Prism for Windows (GraphPad Software, San Diego California, USA).

## Results

### IL-4/IL-4R subunit expression in cartilage

**Figure 1:**
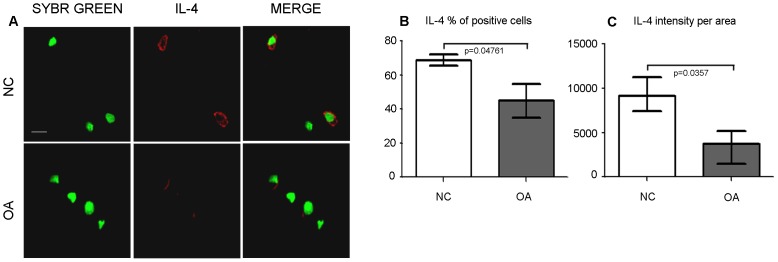
Analysis of IL-4 expression in normal and osteoarthritic cartilage. **A**: representative examples of IL-4 staining in controls (NC), and OA cartilage as detected by confocal microscopy. Panels show IL-4 staining (Dy Light 647, rendered in red), nuclear counterstaining (sybr green, rendered in green) and merged signals (bar  = 10 µm). **B**: percentage of IL-4 positive cells in mid-deep layers of control (NC, n = 3), and OA cartilage (n = 6). Bars and whiskers indicate mean and S.E.M., respectively. Data were compared with unpaired data analysis using the Mann-Whitney U test (p = 0.0476). **C**: level of IL-4 expression (shown as mean intensity per positive cell area) in controls (NC), and OA cartilage following confocal analysis. Comparison was performed by the Mann-Whitney U test (p = 0.0357).

IL-4 expression was evaluated using confocal microscopy in frozen sections of cartilage (NC n = 3, OA n = 6). A representative example of IL-4 staining is shown in Figure 1, panel A. A small number (less than 10%) cells in the superficial layer were IL-4 positive. In the mid/deep layers, the percentage of IL-4 positive cells was significantly higher (p = 0.0476) in normal cartilage (mean±SEM: 68.7%±3.3%) than OA cartilage (44.9%±9.86%), (Figure 1, panel B). Furthermore, the level of IL-4 expression evaluated as mean intensity per positive cell area was also significantly higher (p = 0.0357) in controls (n = 3) than in OA (n = 5) cartilage (Figure 1, panel C). Notably, one OA case was completely negative, and therefore was excluded from the latter comparison.

**Figure 2:**
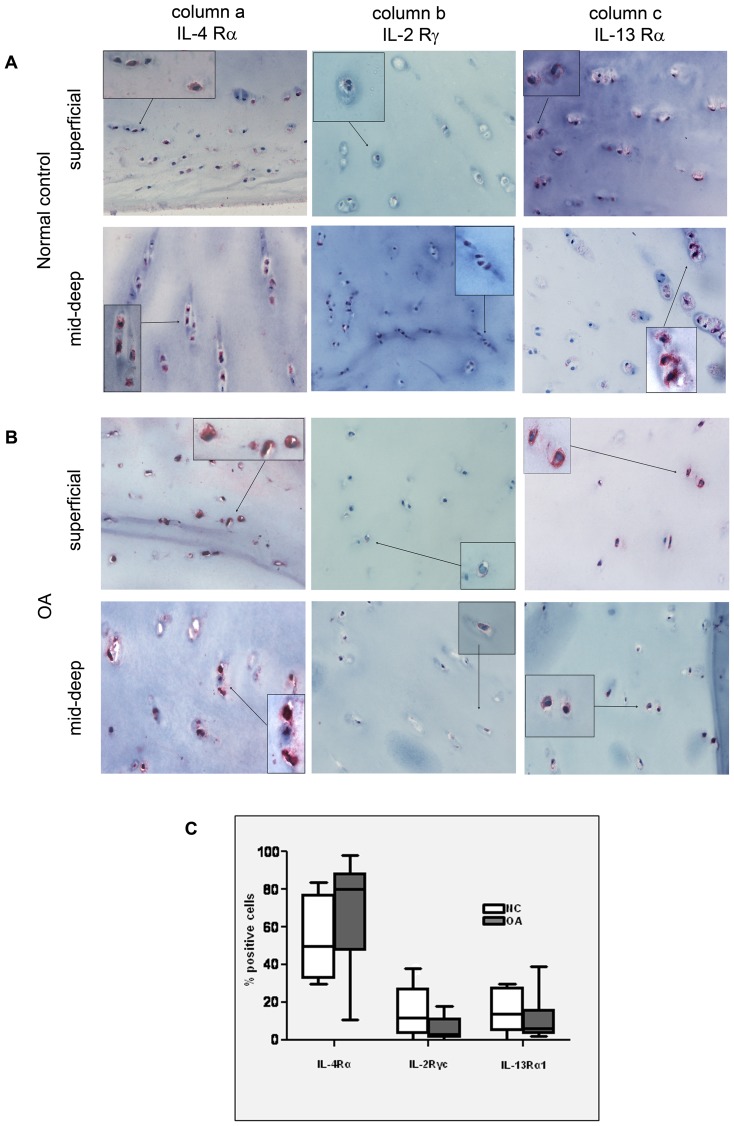
Analysis of IL-4R subunits in normal and osteoarthritic cartilage. **A** and **B**: representative examples of IL-4R subunits staining in control (NC, **A**) and OA (**B**) cartilage (original magnification ×100) with details of superficial and mid-deep layer. Insets: higher magnification views (original magnification ×200). **C**: Percentage of positive cells expressing the three IL-4R subunits in controls (NC, white pattern, n = 5), and OA cartilage (dark pattern, n = 6), in the mid-deep layers. Comparison of NC and OA data was performed by the Mann-Whitney U test (p = ns). Boxes indicate the 25% and 75% percentiles, whiskers indicate the minimum to maximum values, and bars indicate the median.

Immunohistochemistry was used to assess the percentages of IL-4R subunit positive cells across the cartilage layers. Chondrocytes of both normal and OA articular cartilage expressed the three receptor chains. Positive staining for the IL4-Rα, IL13-Rα1 and the IL-2Rγc were found in chondrocytes in all zones of normal or OA cartilage specimens, but with a much higher prevalence of positive cells in the mid/deep layers (Figure 2A and 2B). Therefore, at the level of the latter cartilage zone, the percentages of positive cells over the total number of cells were counted and are shown in Figure 2C. Notably, OA cartilage samples presented a trend toward a lower percentage of both IL-2Rγc and IL-13Rα1 positive cells in the mid/deep layers (Figure 2C), thus suggesting a reduced ability to assemble full functional receptor. No statistical differences were found in the number of cells found to be positive for different IL-4R subunits between control and OA samples (Fig. 2C).

**Figure 3:**
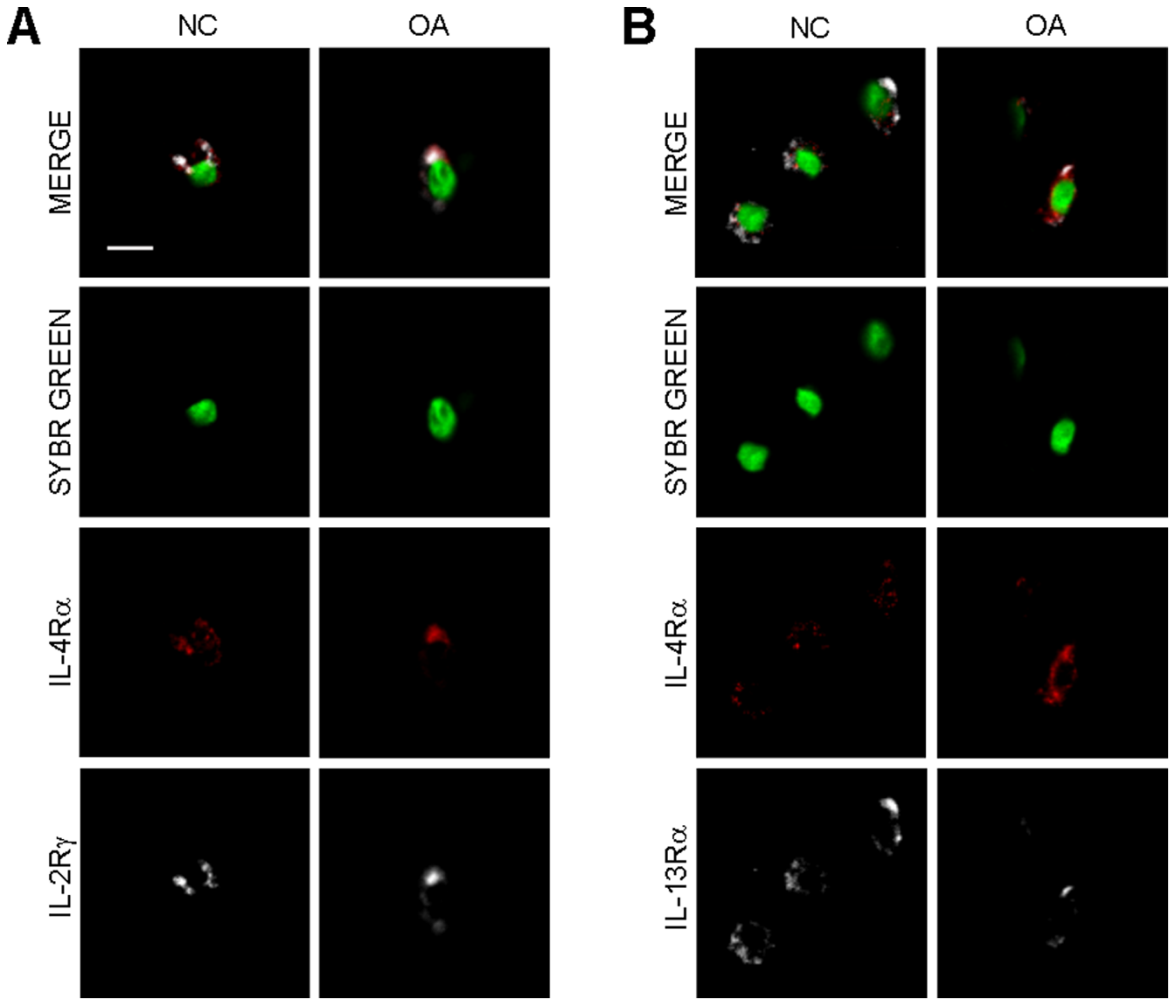
Co-localization of IL-4R subunits expressed in normal and OA cartilage. **A**: a representative example of IL-4Rα and IL-2Rγ colocalization (IL-4 Receptor type I) in control and OA cartilage. **B**: a representative example of IL-4Rα and IL-13Rα1 (IL-4 Receptor type II) in control and OA cartilage (bar  = 10 µm). Panels show IL-4R staining (Dy Light 647, rendered in red), either IL-2Rγ or IL-13Rα (Alexa 555, rendered in white), nuclear counterstaining (sybr green, rendered in green) and merged signals.

To further investigate which type of IL-4 receptor is present in OA cartilage and controls, co-localization of IL-4R subunits was performed (OA n = 3, NC n = 2). In control and OA cartilage, the IL-4Rα chain is associated with either IL-2Rγc or IL13-Rα1 to form Type I or Type II IL-4 receptors as indicated by the merged signals (upper row of Figures 3A and 3B).

### Evaluation of the maintenance of a properly differentiated phenotype and IL-4R subunits expression in high-density chondrocytes

**Figure 4:**
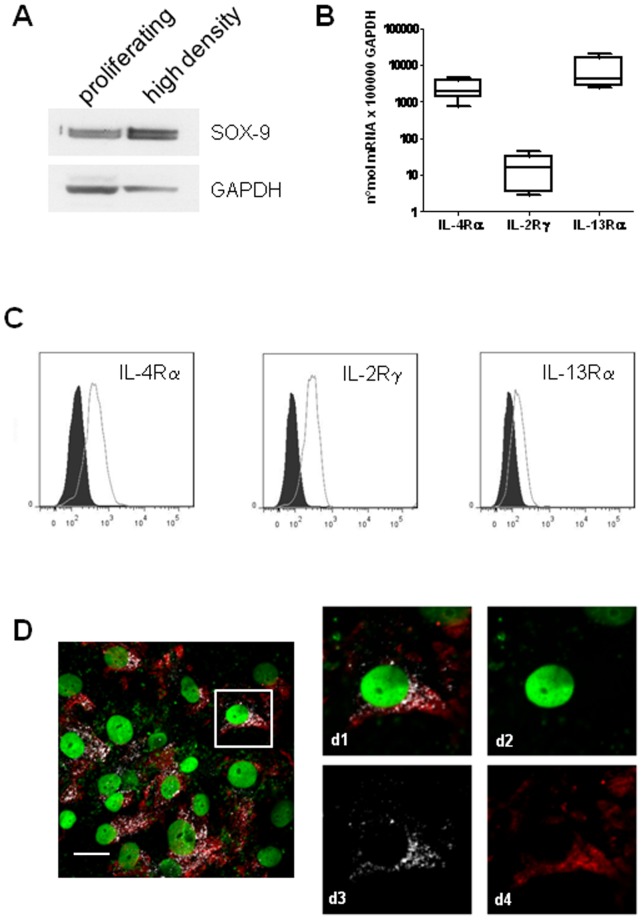
Assessment of the maintenance of a differentiated phenotype and evaluation of IL-4R subunits repertoire by high density cultures of primary chondrocytes. A: SOX-9 protein expression in high density cultures compared to proliferating chondrocytes by western blot analysis. The housekeeping protein GAPDH is used as a loading control. A representative example out of the four examined. B: Cumulative evaluation of IL-4 receptor subunit gene expression in high-density chondrocytes in 5 different patients. Values represent the number of molecules per 100,000 GAPDH as assessed by real time PCR. Boxes indicate the 25% and 75% percentiles, whiskers indicate the minimum to maximum values, and bars indicate the median. C: flow cytometric analysis of the level of expression of each IL-4R subunit in high density culture OA chondrocytes. Dark pattern: isotype control; white pattern: Dy Light 647 signal referring to IL-4Rα (left), IL-2Rγ (middle) and IL-13Rα (right). Each curve refers to 5,000 cells. D representative example of IL-4Rα and IL-2Rγ colocalization (IL-4 Receptor type I) in high-density cultures of OA chondrocytes (bar  = 25 µm). On the right, higher magnifications showing IL-4R staining (Dy Light 647, rendered in red in d4), IL-2Rγ (Alexa 555, rendered in white in d3), nuclear counterstaining (sybr green, rendered in green in d2) and merged signals (d1).

Compared to log phase p1 chondrocytes, high-density cultures expressed a higher protein level of SOX-9 (Fig 4A), the master chondrocytic transcription factor which drives the expression of collagen 2B and aggrecan and is therefore considered to be a marker of differentiated chondrocyte phenotype [35]. The differentiated phenotype was maintained in the cultures because of the high-density seeding [36]. The status of differentiated chondrocytes was also confirmed by evaluating the pattern of IL-4 receptor subunits: real time PCR analysis indicated that high-density cultures expressed transcripts encoding IL-4Rα, IL-2R common γ chain and IL-13Rα1 (Figure 4B) and therefore these cultures were indeed “primary” according to Guicheux and coworkers [7]. Expression of the three chains was also confirmed at the protein level by flow cytometry, showing a much stronger expression of the chains constituting IL-4R type I, i.e. IL-4Rα and IL-2Rγ (Figure 4C). Confocal analysis confirmed the presence of a high level of IL-4Rα and the common γ chain, with a certain degree of colocalization, thus suggesting the preferential usage of the type I receptor, a feature of differentiated chondrocytes, by chondrocytes cultured in this *in vitro* model. (Figure 4D).

### IL-4 modulation on IL-1β-stimulated OA chondrocyte production of chemokines and ECM-degrading enzymes

#### 1) Chemokines

**Figure 5:**
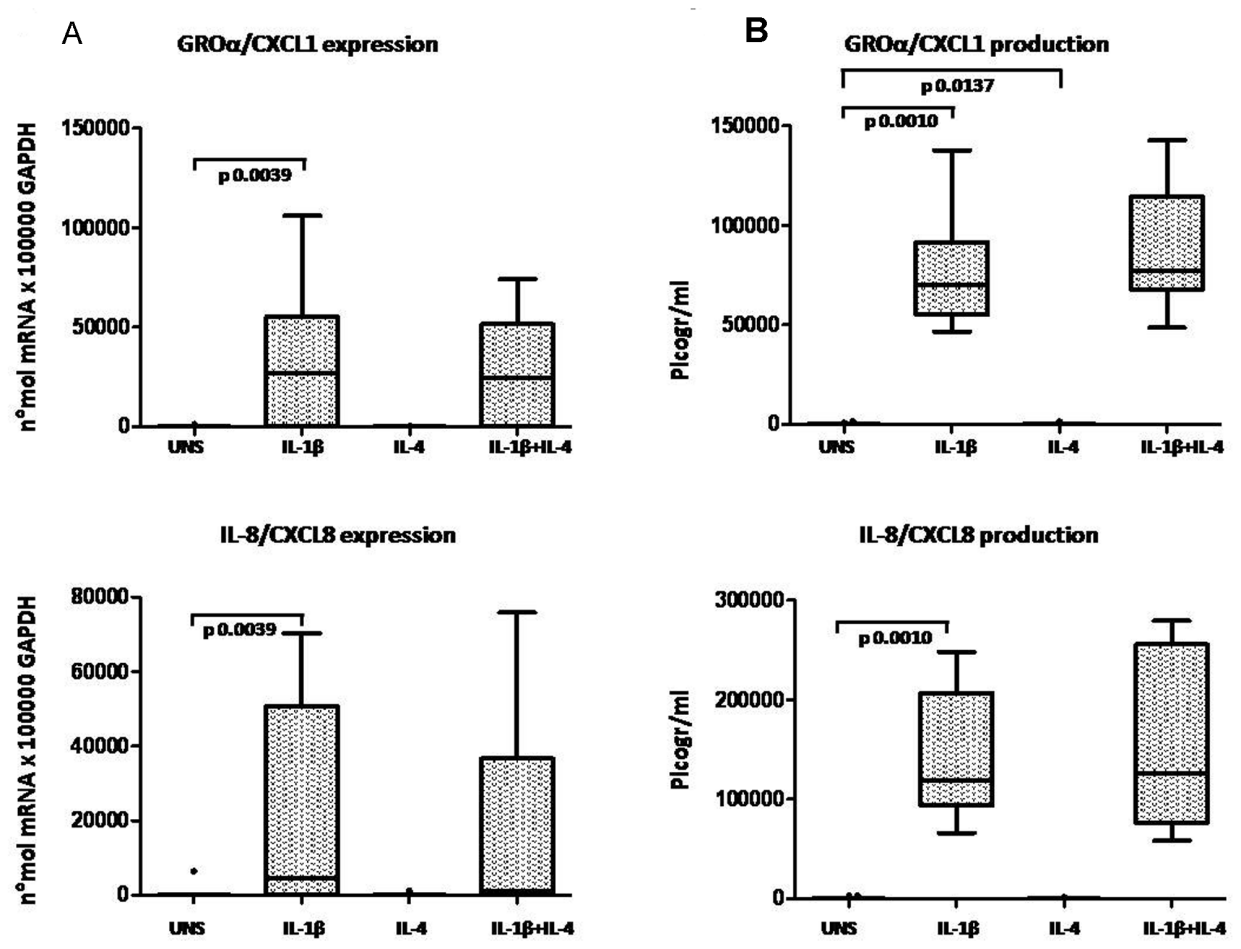
Evaluation of IL-4 anti-inflammatory activity on CXC chemokine expression in high-density OA chondrocytes. **A**: mRNA expression; **B**: protein production. Chemokines were detected in chondrocytes (cells and culture supernatants) after 24 hours of incubation in unstimulated condition (UNS) and in the presence of IL-1β (2 ng/ml), IL-4 (10 ng/ml), or a combination of the two cytokines. Boxes indicate the 25% and 75% percentiles, whiskers indicate the minimum to maximum values, bars indicate the median; solid circles indicate outliers. After Bonferroni's correction, p values below 0.0165 were considered significant.

**Figure 6:**
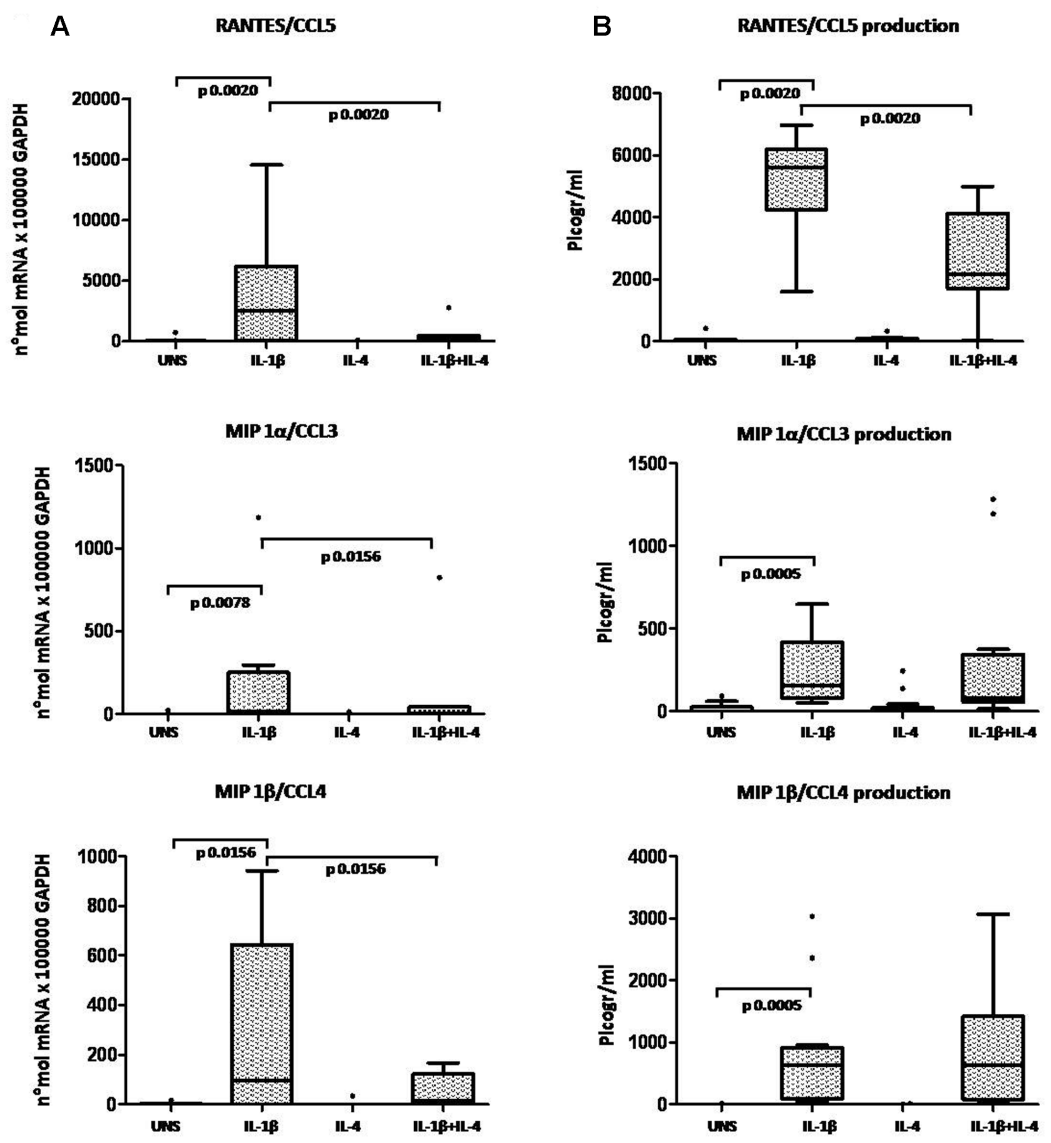
Evaluation of IL-4 anti-inflammatory activity on CC chemokine expression in high-density OA chondrocytes. **A**: mRNA expression; **B**: protein production. Chemokines were detected in chondrocytes (cells and culture supernatants) after 24 hours' incubation in unstimulated conditions (UNS) and in the presence of IL-1β (2 ng/ml), IL-4 (10 ng/ml), or a combination of the two cytokines. Boxes indicate the 25% and 75% percentiles, whiskers indicate the minimum to maximum values, bars indicate the median; solid circles indicate outliers. After Bonferroni's correction, p values below 0.0165 were considered significant.

OA chondrocytes were analyzed for chemokine mRNA expression. Unstimulated and IL-4 treated chondrocytes expressed low levels of chemokine mRNA when analyzed by a sensitive Real Time PCR assay. In response to 24 h stimulation with IL-1β, chemokine mRNA was up-regulated in all the high-density chondrocyte cultures analyzed. Chondrocyte co-stimulation with IL-1β and IL-4 showed that the presence of IL-4 did not affect GROα/CXCL1 and IL-8/CXCL8 mRNA expression induced by IL-1β (Figure 5A). Conversely, IL-4 significantly inhibited RANTES/CCL5 (p = 0.0020), MIP-1α/CCL3 (p = 0.0156) and MIP-1β/CCL4 (p = 0.0156) mRNA expression induced by IL-1β (Figure 6A).

ELISA was performed on chondrocyte culture supernatants to investigate the IL-4 modulation of IL-1β-induced chemokine production at protein levels. IL-1β induced secretion of GROα/CXCL1 and IL-8/CXCL8 protein was unaffected by co-stimulation with IL-4 (Figure 5B). Conversely, IL-4 significantly inhibited IL-1β-induced secretion of RANTES/CCL5 (p = 0.0020), in keeping with the effects on mRNA expression, whereas MIP-1α/CCL3 (p = 0.1909) and MIP-1β/CCL4 (p = 0.2661) chemokine production was not significantly modulated by IL-4 (Figure 6B).

#### 2) Matrix Degrading Enzymes and inhibitors

**Figure 7:**
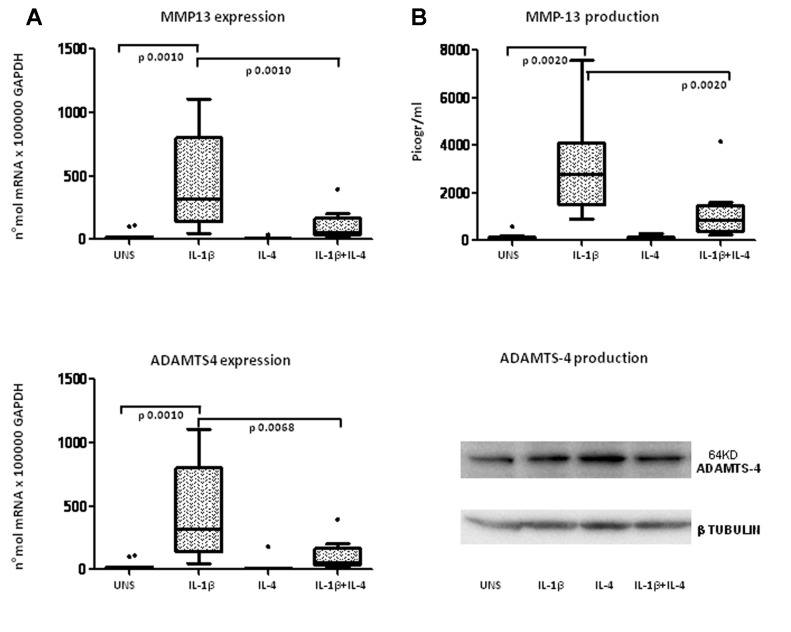
Evaluation of IL-4 anti-inflammatory activity on extracellular matrix-degrading enzymes (MMP-13 and ADAMTS4) in high-density OA chondrocytes. A: mRNA expression; B: protein production. MMP-13 was detected in chondrocytes (cells and culture supernatants) after 24 hours of incubation in unstimulated conditions (UNS) and in the presence of IL-1β (2 ng/ml), IL-4 (10 ng/ml), or a combination of the two cytokines. Whereas ADAMTS4 mRNA was detected as for the other transcripts, ADAMTS4 protein was assessed in lysates of cells scraped with their extracellular matrix. WB show the band at 64 kDA along with β-tubulin as a loading control. Boxes indicate the 25% and 75% percentiles, whiskers indicate the minimum to maximum values, bars indicate the median; and solid circles indicate outliers. After Bonferroni's correction, p values below 0.0165 were considered significant.

To understand whether IL-4 is able to modulate ECM remodeling in response to pro-inflammatory stimuli, we evaluated the RNA and protein expression of major matrix degrading enzymes and their inhibitors. This analysis showed that IL-4 down regulated the IL-1β-induced mRNA expression of MMP-13 (p = 0.0010, Figure 7A) (confirmed by ELISA measurement of the protein: p = 0.0020, Figure 7B) and ADAMTS-4 (p = 0.0068, Figure 7A). ADAMTS-4 protein expression was not affected by the treatments, as evaluated by western blotting of lysates of cells together with their ECM (one representative example out of four examined is shown in Figure 7B, lower panel).

Finally, neither IL-1β nor IL-4 appeared to significantly modulate ADAMTS-5, TIMP-1, TIMP-3 mRNA expression (data not shown).

Experiments performed with control non-OA chondrocytes (n = 3) showed similar effects of IL-4 with regard to the modulation of the IL-1β-stimulated production of chemokines and matrix degrading enzymes (data not shown).

## Discussion

### Expression of the chondroprotective cytokine IL-4 is reduced in OA cartilage

Published data reports IL-4 expression in both healthy and OA cartilage [14], but no quantitative information is available concerning the differential expression of IL-4 protein or its receptors.

The main aim of this study was therefore to analyze IL-4 expression in cartilage from OA and normal tissues by taking into account both the percentage of positive cells and the level of expression in each cell, obtained using confocal microscopy. IL-4-positive cells were mainly detected in cartilage mid-deep layers, in keeping with depth-dependent variations in mechanotransduction and hydrostatic pressure loading [37]. It is noteworthy that a significantly lower percentage of IL-4-positive cells were detected in these cartilage zones in OA samples. Furthermore, confocal quantitative analysis showed, for the first time, that IL-4 expression is significantly impaired in OA cartilage. Collectively, our data support the hypothesis that changes in IL-4 levels might be involved in OA pathophysiology.

### Both OA and non–OA articular cartilage express all the three IL-4 receptor subunits (IL-4Rα, IL-13Rα1 and γ chain) that assemble to yield both the type I and the type II multimeric IL-4 receptors

To further explore the reasons for the impairment of the chondroprotective IL-4 pathway in OA we performed an immunohistochemical analysis to evaluate the percentage expression of each IL-4 receptor subunit (IL-4Rα, IL-13Rα1 and γ chain) in either superficial or mid/deep layers of both normal and OA cartilage samples. Our data show that IL-4R subunit expression in terms of percentages of positive cells is not significantly different in OA patients compared to controls, in keeping with previously published data [12]. Only a few cells in the superficial layer of cartilage expressed IL-4Rα, γ chain and IL-13Rα1, whereas in mid/deep cartilage layers expression of these subunits was much higher, and OA samples always expressed a lower, albeit not significantly, percentage of positive cells compared to control tissue, particularly in the case of γ chain.

Previous data reported the expression of these three subunits in human OA and normal cartilage [12], but little is known about the type of functional multimeric receptor complex used by chondrocytes. To investigate this issue, Millward-Sadler at al exploited an “IL-4 crosslinking” approach followed by western blotting and concluded that chondrocytes from normal and OA cartilage signal through a Type II IL-4R. However, in their experimental approach they delivered IL-4 to chondrocytes “in suspension”, which is not an optimal setting for cytokine stimulation of chondrocytes [38], which requires focal adhesions and sensing the extracellular matrix [39]. It might be conceivable that upon IL-4 binding to IL-4Rα, γ-chain recruitment is more affected by the absence of ECM proteins and therefore of focal adhesion complexes compared to IL-13Rα1 recruitment. Conversely, to investigate the occurrence of heterodimerization of IL-4Rα with either γc or IL-13Rα1, we used a confocal microscopy co-localization approach of the two chains, which is highly suggestive of dimerization. Our finding indicated that both IL-4R type I and IL-4 R type II are present in cartilage tissue.

### IL-4 shows differential abilities to abrogate the IL-1β induction of chemokines and matrix-degrading enzymes in human differentiated chondrocytes

Several studies, mainly carried out in animal tissues, have shown that chondrocyte response to IL-4 might be considered chondroprotective [6], [8], [9], [40], [41], [42]. Little is currently known, however, regarding the ability of IL-4 to provide a protective, anti-inflammatory action in human cartilage. To undertake this section of the study we adopted a high-density culture model of primary chondrocytes, used within the first passages as a universally accepted method to regain a differentiated phenotype [36], [43]. This was confirmed by SOX-9 protein expression and expression of all the IL-4 receptor subunits, particularly the IL-2R γ chain at both RNA and protein levels. The discrepancy between the level of gene and protein expression of the IL-4R subunits in culture, particularly for IL-13Rα1, might be due to a substantial production of the soluble form of the IL-13Rα1 [44]. γ chain has been previously linked to a “differentiated primary” chondrocyte phenotype [7] but also to non-responsiveness of chondrocyte cultures or cartilage explants to IL-4. Since our data conversely indicate that IL-4 is able to modulate selected IL-1β-induced genes and some of them both at mRNA and protein levels, we argue that the differences might be due to different experimental conditions: IL-4 (50 ng/ml) pretreatment for 48 hours prior to IL-1β (1 ng/ml) addition for 72 hours in [7] versus IL-4 (10 ng/ml) added at the same time with IL-1β (2 ng/ml) and left for 24 hours in our experimental settings.

In this study, we analyzed the effect of IL-4 on the expression of several inflammatory/catabolic mediators induced by IL-1β in human OA chondrocytes. In particular, we focused on the ability of IL-4 to modulate the production of soluble factors belonging to the CXC and CC chemokine subfamilies, ECM-degrading enzymes and TIMPs. All these mediators are recognized as being highly involved in cartilage remodeling [2], [45].

IL-1β stimulation of human chondrocytes induced a promptly and markedly increased mRNA expression of a large set of chemokines and, among these, IL-8/CXCL8 and GROα/CXCL1 appear to be the most highly regulated [32]. IL-8/CXCL8 and GROα/CXCL1 have been shown to alter the chondrocyte phenotype by inducing hypertrophic differentiation [33], [46]. IL-4, unable to hamper the expression and production of these early and strongly regulated CXC chemokines, appears to be ineffective in preventing this key event in OA pathogenetic pathways. Conversely, IL-4 was shown to down-regulate the IL-1β-induced gene expression of the CC chemokines RANTES/CCL5, MIP-1α/CCL3, MIP-1β/CCL4.

To date, very few published data are available concerning IL-4 activity on inflammatory chemokine expression and production by the different cell types of the joint compartments. Available studies indicate different effects of IL-4 depending on the specific gene, stimulus and cell type, thus raising the possibility that IL-4 effects might even be species and tissue-specific [47], [48]. Overall, our results confirm the differential suppressive efficacy of IL-4 on chondrocyte chemokine expression as a function of the specific gene product and its transcriptional control. Further studies are needed to detail STAT6 ability to affect chemokine transcriptional control in human chondrocytes. Indeed, in most of the cellular models considered to date, the IL-4-dependent regulation of inflammatory gene expression has been mainly attributed to the STAT6 transcription factor [20], [49], [50]. The promoter region of the human chemokine genes harbors putative binding sites for a number of well-described transcription factors (e.g: C/EBP, NF-κB, AP-1, IRF) which vary in their quantitative and qualitative contribution among specific sets of chemokine genes. In chondrocytes, recent evidence has shown that the C/EBP binding site is highly represented and overcomes the NF-κB binding site in a set of IL-1β highly-inducible chemokine genes, including IL-8/CXCL8, GROα/CXCL1, MIP-1α/CCL3, MIP-β/CCL4 [32], [51], whereas in the RANTES gene, binding sites for NF-κB and IRF factors prevail [32], [52], [53], [54].

Bearing these data in mind, we may take into consideration the following explanations.

Firstly, the strong inhibitory effect of IL-4 on IL-1β induced RANTES/CCL5 expression, detectable at both mRNA and protein levels, might be attributable to the ability of STAT6 to interfere with both NF-κB and IRF [50], [55], [56], [57], [58], [59]. This hypothesis is supported by studies performed on other cell systems that indicate both a critical role of NF-κB in RANTES/CCL5 expression [60] as well as IL-4 dependent STAT6 ability of inhibiting RANTES/CCL5 gene transcription [61].

Secondly, the lack of IL-8/CXCL8 and GRO-α/CXCL1 gene modulation by IL-4 may be the result of: a) a weaker ability of STAT-6 mediated mechanisms to interfere with NF-κB elements, due to a higher affinity of NF-κB binding sites present in the promoter region of these genes [62] or to a lower NF-κB relative contribution in promoting gene transcription; b) the presence of synergic binding site response elements other than NF-κB, (C/EBP and MEF-3, both highly expressed in IL-8/CXCL8 and GRO-α/CXCL1 gene promoters, AP etc) that are able to sustain gene transcription rate and maintain the mRNA steady-state even when NF-κB action is down-regulated [32], [62].

Finally, it is intriguing to note that in our study the IL-4 inhibition of MIP-1β/CCL4 and MIP-1α/CCL3 was observed on mRNA expression without detecting a concomitant protein level modification. Since IL-4-mediated gene modulation may occur at early (2–6 h) and late (24–48 h) time points [63], we might suppose that these genes are only modulated late. Therefore, at our experimental time (24 h), IL-4-induced regulation was only detectable at mRNA levels, but was not yet evident at the level of protein production.

Based on this evidence, we may speculate that the ability of IL-4 to down-modulate the different chemokine genes depends on the chondrocyte specific quantitative and qualitative profile of various transcription factor binding sites related to specific signal transduction machinery. It is also noteworthy that IL-8/CCL8, GRO-α/CXCL1, MIP-1α/CCL3 and MIP-1β/CCL4 belong to the secretome of normal chondrocytes [31], [64], [65], possibly supporting the hypothesis of a housekeeping expression level of matrix-degrading activity which is needed for the tightly-regulated, low-homeostatic turnover of ECM proteins. Conversely, RANTES expression only occurs in OA chondrocytes [66] and therefore, from a pathophysiological point of view, the ability of IL-4 to counteract IL-1β-dependent RANTES induction is highly relevant.

We also analyzed the effects of IL-4 on other soluble factors strongly involved in cartilage matrix break-down, namely ECM–degrading enzymes: MMP-13, ADAMTS-4,-5 and tissue inhibitors TIMP-1 and -3. The latter were chosen from among the four TIMPs because TIMP-1 is the most expressed tissue inhibitor in both normal and OA cartilage [67], and TIMP-3 has the unique property of being able to counteract both MMPs and ADAMTS. IL-1β-stimulated gene expression of MMP-13 and ADAMTS-4 was strongly inhibited by IL-4, whereas neither IL-1β nor IL-4 had any effect on ADAMTS-5, TIMP-1 or 3 gene expression. The combined action of IL-4 on proteases active on both the aggrecan and the collagen 2 components of the extracellular matrix is particularly interesting in light of recent findings in OA pathogenesis [26]. A first, potentially reversible step in ECM remodeling, is proteoglycan depletion mediated by the ADAMTS enzymes. This results in the activation of the chondrocyte discoidin domain receptor (DDR-2) through interaction with denuded collagen type II. DDR-2 activation then triggers the production of MMP-13, the true no–return point in OA pathogenesis, being able to sustain the production of collagen 2 neoepitopes, which then positively feed back into ECM remodeling [68].

In OA cartilage, MMP-13 and ADAMTS-4,-5 are therefore the foremost degrading enzymes of collagen II and aggrecan, respectively, and a growing body of evidence underlines their contribution to OA disease [3], [16], [26], [29], [69], [70]. It is worth noting that ADAMTS-4 is selectively over-expressed in human OA cartilage, with a positive correlation with the degree of cartilage destruction, whereas ADAMTS-5 is constitutively expressed in both healthy and OA cartilage [71]. The high IL-1β-inducibility of ADAMTS-4 has been recently detailed at the transcriptional level [72] with very recent findings regarding its post-transcriptional regulation [73]. In this study, we evaluated ADAMTS-4 protein by western blotting in lysates obtained from the high-density cultures together with their extracellular matrix, since it is known that ADAMTS have the unique ability to bind to ECM via their TS domains. Prominent bands at 75 and 65 KD were observed, without any differences across the various conditions. The lack of ADAMTS-4 protein induction upon IL-1β stimulation was previously reported by Pratta and colleagues [74] who hypothesized that IL-1β may act through the activation of a constitutively produced protein rather than by increased protein production. Proteolytic activation of the zymogen results in C-terminal truncation, release of the enzyme from the ECM and alteration of its activity profile [75]. Shorter activated forms are mainly 75 kDa, 60 kDa and 50 kDa in pig tissue [76], but slight variances in these molecular masses have been reported by different research groups [75], [77], [78]. Therefore, most of the ADAMTS-4 protein in our cultures corresponded to the activated form. Time course experiments previously showed that the maximum mRNA level for ADAMTS4 expression is at 24 hours, whereas for MMP13 it is at 12 hours [79]. In our 24-hour experimental design IL-1β stimulation as the best compromise between cytokines and ECM enzyme expression, we saw modulation in MMP13 at both mRNA and protein level, whereas ADAMTS-4 was only modulated at mRNA level. We can speculate that in our experimental setting, chondrocytes already expressed a high ADAMTS-4 protein level most of which was constitutively activated with a lack of any modulation. Interestingly, this was also in keeping with results of western blotting to detect the aggrecan cleavage neoepitope NITEGE (data not shown). IL-1β is able to further stimulate ADAMTS-4 mRNA expression but since this peaks at 24 hours, this stimulation time might be too short to see newly synthesized ADAMTS-4 protein or changes in the amount/type of activated forms. An alternative explanation might be that in our high-density cultures, the regulatory miR-125b [73] might be present at such a high level to achieve a post-transcriptional inhibiting effect on new ADAMTS-4 protein synthesis. This is in keeping with some of our unpublished observations collected with high-density long-term chondrocyte cultures, which indicate miR-125b as being among the most highly expressed miR (Agilent genome wide microRNA expression chip, unpublished data from Borzì R.M.). The post-transcriptional inhibiting effect of high miR-125b level on new ADAMTS-4 synthesis might instead fail in conditions of chronic inflammation as recently reported [73] and in those conditions the protective effect of IL-4 might be more easily evident.

## Conclusions

This is the first report to suggest a differential expression of IL-4 in OA versus non-OA cartilage in terms of percentage of positive cells and level of expression. Among a large set of soluble factors (chemokines, ECM degrading enzymes and TIMPs) implicated in pathways leading to cartilage loss, we have reported the ability of IL-4 to hamper markedly IL-1β-induced mRNA and protein expression of RANTES/CCL5 and MMP-13, whereas MIP-1α, MIP-1β and ADAMTS-4 were only modulated at mRNA level. Differences in the spectrum of IL-4 biological effects might be due to differences in the transcriptional and posttranscriptional control of the selected genes. Since the expression of RANTES/CCL5 and MMP-13 is a specific feature of OA articular chondrocytes and these proteins have no housekeeping function in normal healthy articular cartilage, our data further underline the potential usefulness of IL-4 treatment in OA.
