# Correction: Human Osteoarthritic Cartilage Shows Reduced *In Vivo* Expression of IL-4, a Chondroprotective Cytokine that Differentially Modulates IL-1β-Stimulated Production of Chemokines and Matrix-Degrading Enzymes *In Vitro*

**DOI:** 10.1371/journal.pone.0105819

**Published:** 2014-08-08

**Authors:** The PLOS ONE Staff

The ninth and tenth authors, Rosa Maria Borzì and Riccardo Meliconi, should be noted as contributing equally to this work.
